# Supplementary material for: Gender, status, and team interaction: A microdynamic exploration of wearable sensor data across 11 research groups
Source: PLoS One. 2026 May 15;21(5):e0349195. doi: 10.1371/journal.pone.0349195 (PMC13178976; doi:10.1371/journal.pone.0349195)

**S1 – Supplementary Tables and Network Statistics**

Table 1 Descriptive statistics of main socio-demographic variables and aggregated categorical variables of team members

| **Characteristic** | **Team-1**  N = 8^1^ | **Team-10**  N = 8^1^ | **Team-2**  N = 10^1^ | **Team-3**  N = 8^1^ | **Team-4**  N = 9^1^ | **Team-5**  N = 11^1^ | **Team-6**  N = 10^1^ | **Team-7A**  N = 7^1^ | **Team-7B**  N = 7^1^ | **Team-8**  N = 8^1^ | **Team-9**  N = 17^1^ |
| --- | --- | --- | --- | --- | --- | --- | --- | --- | --- | --- | --- |
| Gender |  |  |  |  |  |  |  |  |  |  |  |
| Woman | 3 | 5 | 7 | 5 | 5 | 5 | 3 | 2 | 3 | 2 | 6 |
| Man | 5 | 3 | 3 | 3 | 4 | 6 | 7 | 5 | 4 | 6 | 11 |
| Mean age | 45.9 (15.1) | 31.8  (6.9) | 34.0  (5.4) | 35.8 (13.3) | 34.6 (8.9) | 28.6 (4.5) | 38.5 (10.4) | 31.9 (6.8) | 31.0  (6.0) | 30.1 (5.8) | 31.4 (5.1) |
| Age Groups |  |  |  |  |  |  |  |  |  |  |  |
| Elder | 3 | 0 | 0 | 2 | 1 | 0 | 2 | 0 | 0 | 0 | 0 |
| Middle | 3 | 4 | 8 | 3 | 3 | 3 | 5 | 4 | 4 | 3 | 8 |
| Young | 2 | 4 | 2 | 3 | 5 | 8 | 3 | 3 | 3 | 5 | 9 |
| Mean team tenure in months | 61.6 (60.1) | 46.8 (35.7) | 18.7 (18.9) | 38.3 (39.9) | 55.4 (34.9) | 12.0 (11.0) | 31.9 (13.9) | 32.0 (24.0) | 42.6 (29.7) | 29.4 (36.4) | 41.5 (38.6) |
| Members Tenure |  |  |  |  |  |  |  |  |  |  |  |
| Long-term | 5 | 5 | 3 | 2 | 5 | 2 | 9 | 5 | 4 | 4 | 9 |
| Consolidated | 2 | 2 | 0 | 2 | 3 | 0 | 0 | 0 | 1 | 1 | 4 |
| Newcomer | 1 | 1 | 7 | 4 | 1 | 9 | 1 | 2 | 2 | 3 | 4 |
| Seniority |  |  |  |  |  |  |  |  |  |  |  |
| Senior | 4 | 2 | 3 | 2 | 3 | 1 | 4 | 4 | 5 | 3 | 3 |
| Junior | 4 | 6 | 7 | 6 | 6 | 10 | 6 | 3 | 2 | 5 | 14 |
| ^1^n; Mean (SD) | | | | | | | | | | | |

Table 2 Absolute face-to-face detection count by team and organisation

| **Team** | **Face-to-face detections** |  | **Type of organisation** | **Organisation** | **Face-to-face detections** |
| --- | --- | --- | --- | --- | --- |
| Team_1 | 1,493 |  | University | Private company | 16,363 |
| Team_2 | 8,674 |  | Research lab | Research labs | 33,063 |
| Team_3 | 6,545 |  | University | University | 11,419 |
| Team_4 | 2,681 |  | Research lab |  |  |
| Team_5 | 12,772 |  | Research lab |  |  |
| Team_6 | 3,381 |  | University |  |  |
| Team_7A | 7,818 |  | Private company |  |  |
| Team_7B | 7,597 |  | Private company |  |  |
| Team_8 | 948 |  | Private company |  |  |
| Team_9 | 7116 |  | Research lab |  |  |
| Team_10 | 1,820 |  | Research lab |  |  |

#### Team 1 face-to-face interaction network (University)

| Team | Edge-IDs | Detections |
| --- | --- | --- |
| Team_1 | 34-35 | 292 |
| Team_1 | 34-37 | 269 |
| Team_1 | 36-37 | 243 |
| Team_1 | 33-34 | 181 |
| Team_1 | 32-37 | 170 |
| Team_1 | 34-36 | 117 |
| Team_1 | 33-35 | 96 |
| Team_1 | 32-33 | 54 |
| Team_1 | 33-37 | 48 |
| Team_1 | 31-37 | 23 |


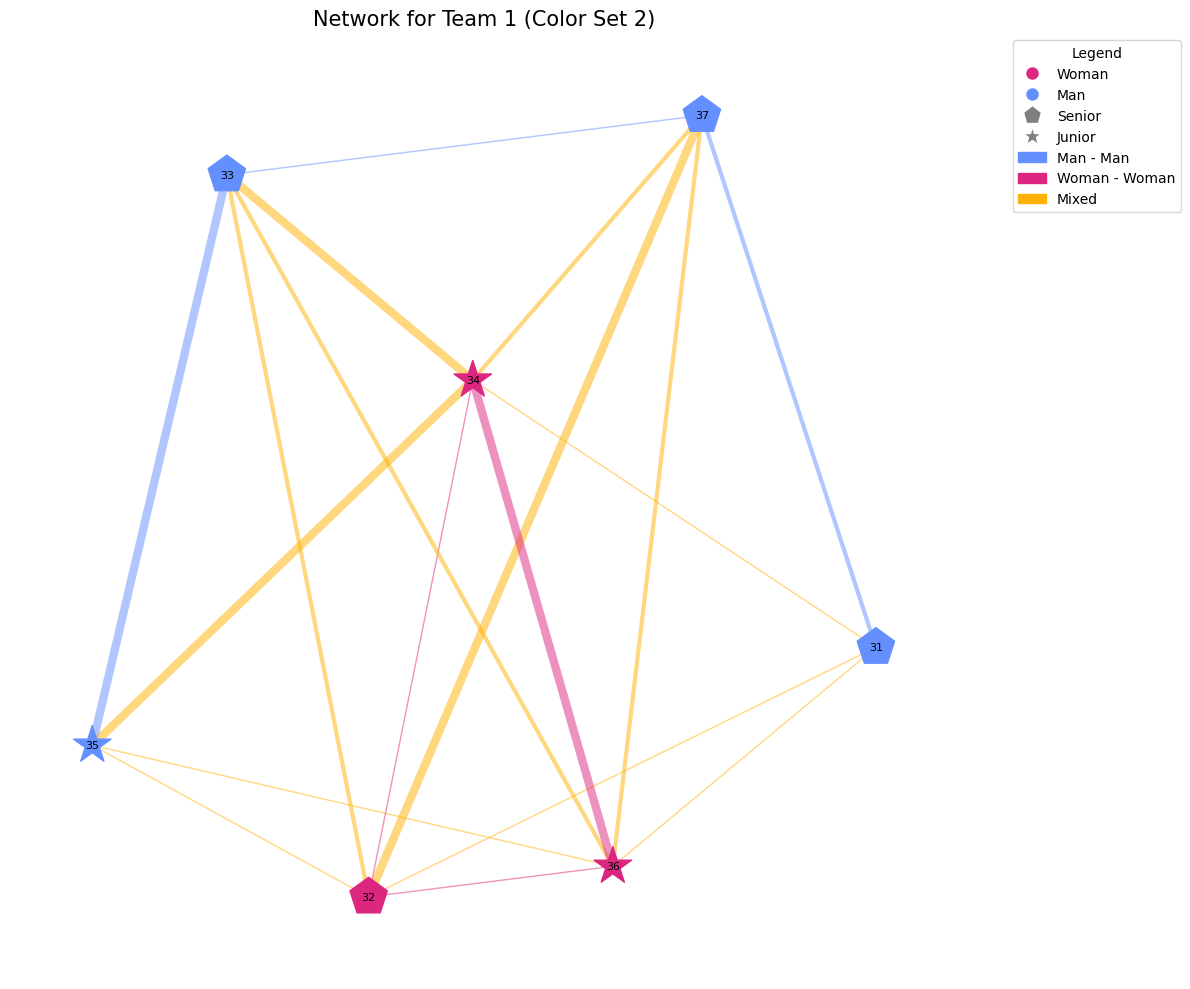


#### Team 2 face-to-face interaction network (Research lab)

| Team | Edge-IDs | Detections |
| --- | --- | --- |
| Team_2 | 3-7 | 1,327 |
| Team_2 | 2-5 | 1,284 |
| Team_2 | 4-7 | 604 |
| Team_2 | 3-5 | 566 |
| Team_2 | 1-2 | 558 |
| Team_2 | 10-2 | 553 |
| Team_2 | 5-9 | 412 |
| Team_2 | 1-5 | 331 |
| Team_2 | 3-9 | 286 |
| Team_2 | 1-3 | 281 |


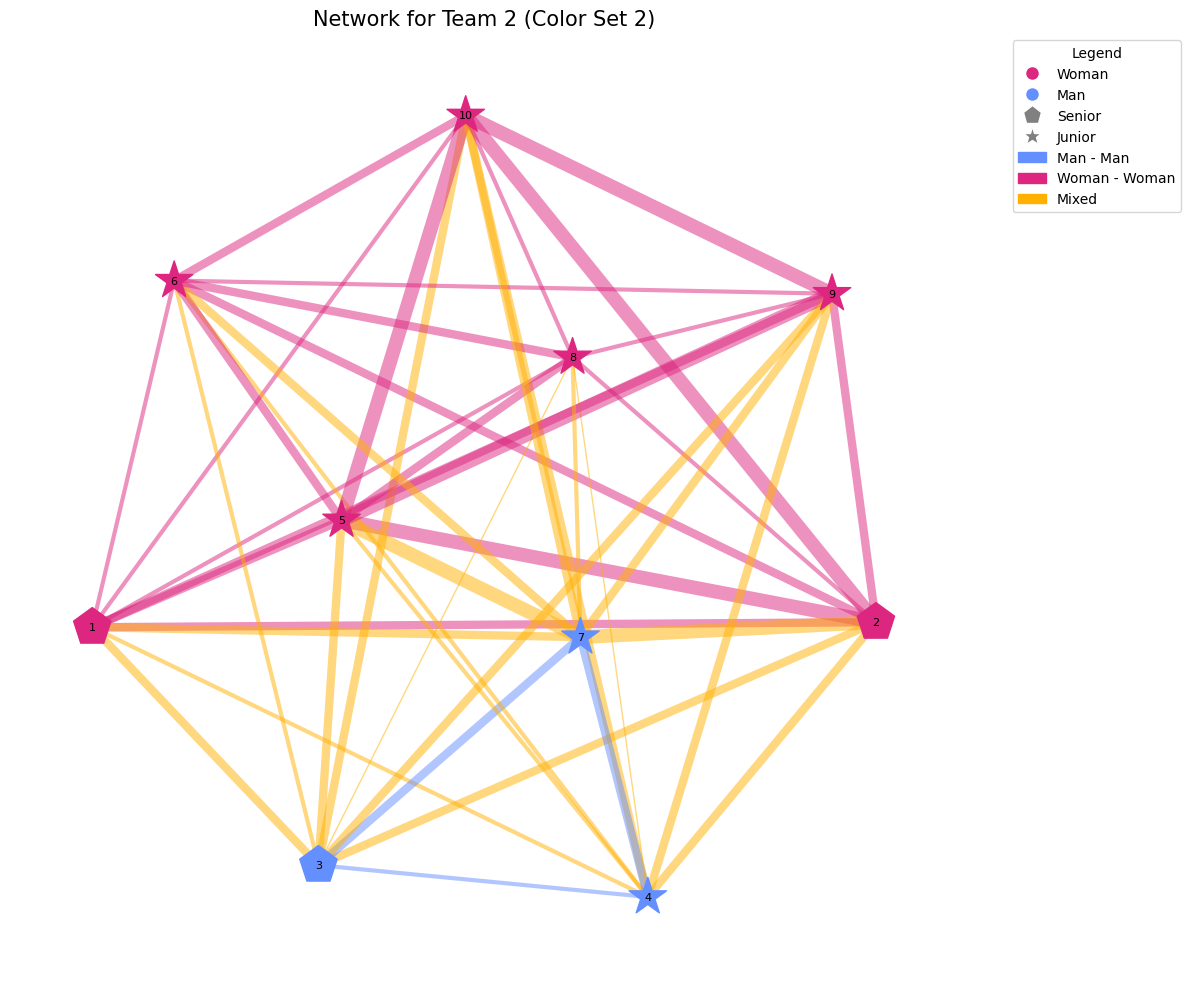


#### Team 3 face-to-face interaction network (University)

| Team | Edge-IDs | Detections |
| --- | --- | --- |
| Team_3 | 53-54 | 3,264 |
| Team_3 | 49-53 | 2,414 |
| Team_3 | 50-53 | 366 |
| Team_3 | 50-51 | 148 |
| Team_3 | 49-54 | 111 |
| Team_3 | 49-52 | 91 |
| Team_3 | 49-50 | 46 |
| Team_3 | 49-51 | 34 |
| Team_3 | 54-55 | 18 |
| Team_3 | 50-52 | 16 |


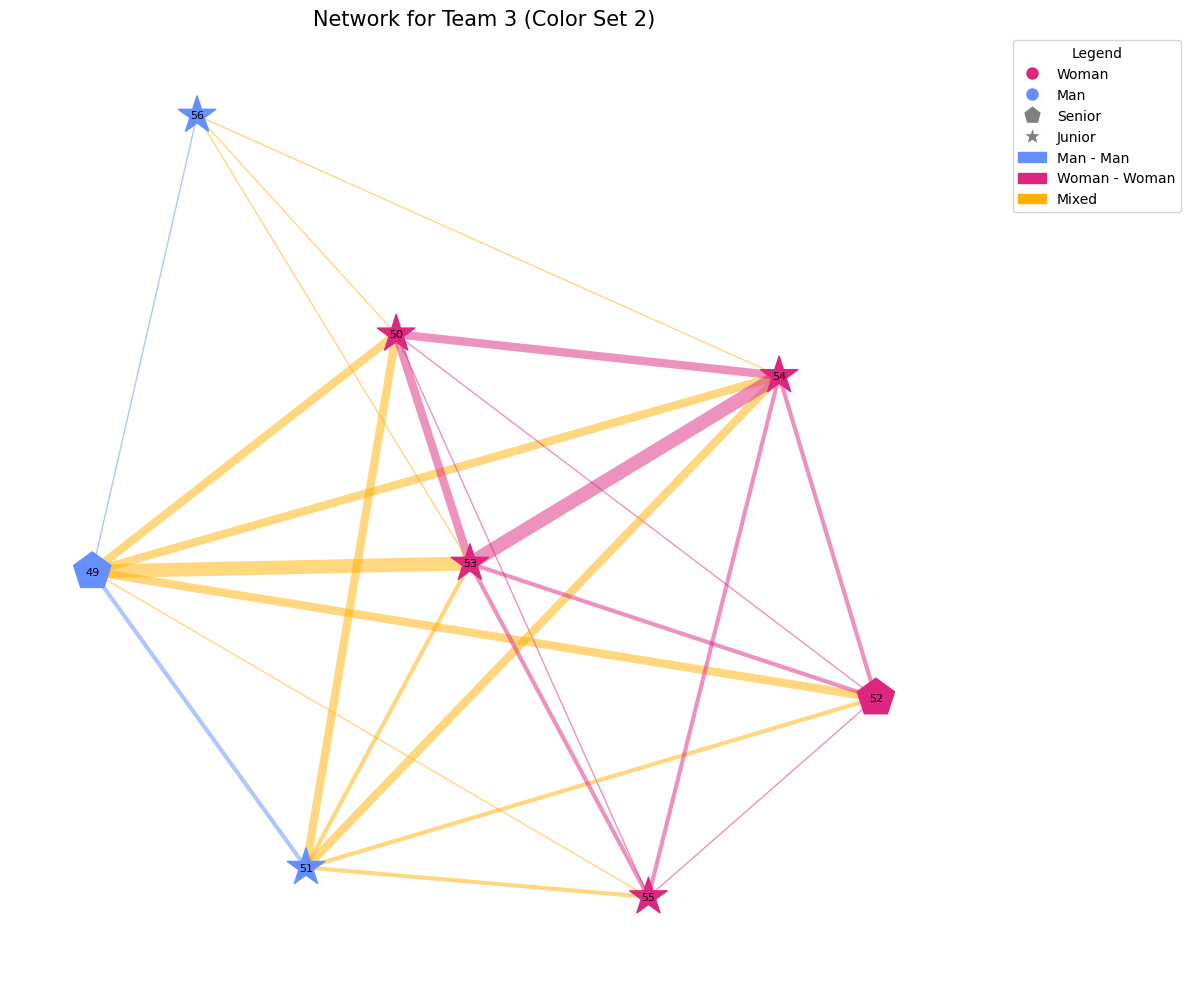


#### Team 4 face-to-face interaction network (Research lab)

| Team | Edge-IDs | Detections |
| --- | --- | --- |
| Team_4 | 28-30 | 482 |
| Team_4 | 26-27 | 350 |
| Team_4 | 25-28 | 336 |
| Team_4 | 25-26 | 217 |
| Team_4 | 27-28 | 171 |
| Team_4 | 22-28 | 170 |
| Team_4 | 24-28 | 158 |
| Team_4 | 26-29 | 135 |
| Team_4 | 26-30 | 127 |
| Team_4 | 22-24 | 118 |


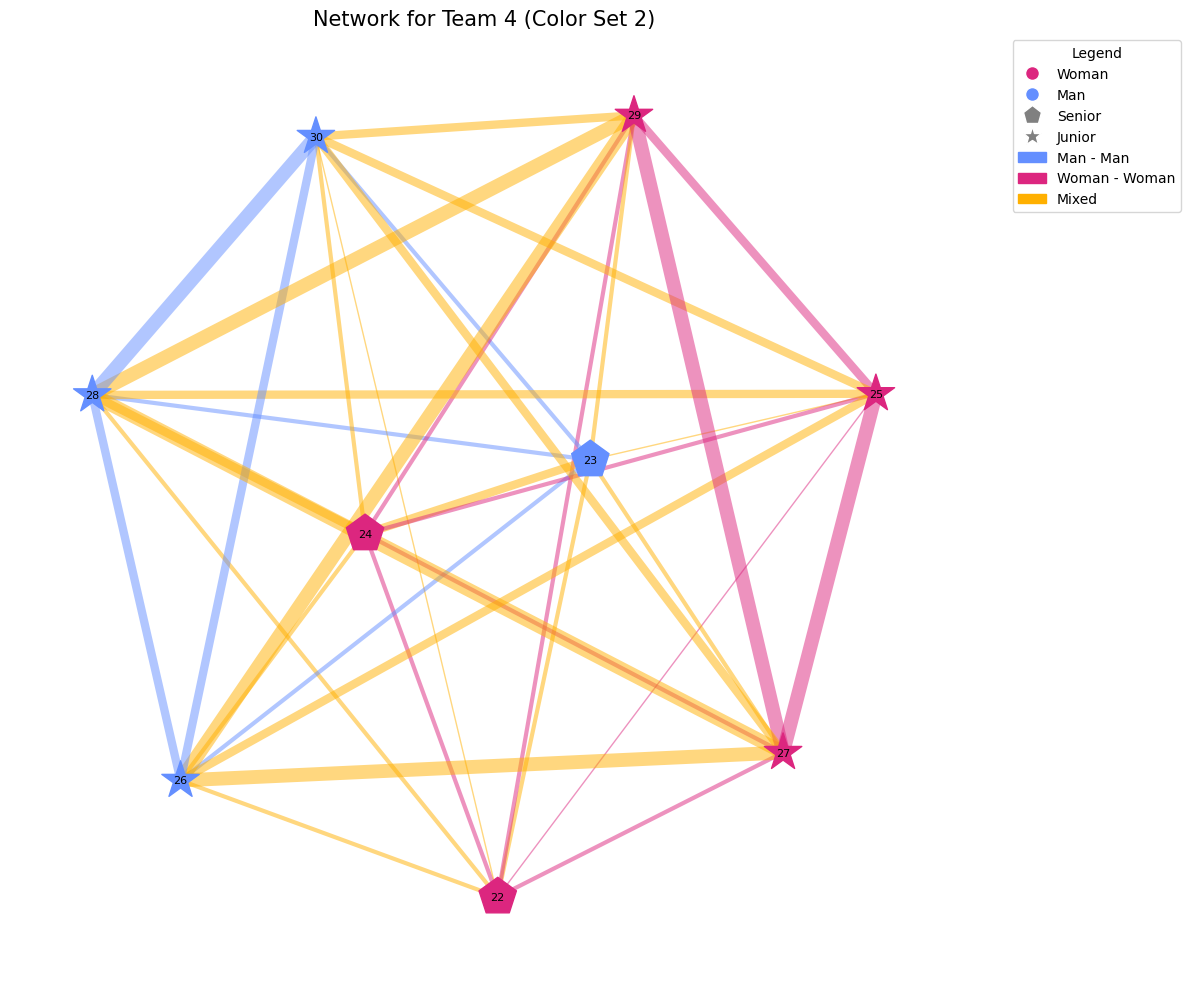


#### Team 5 face-to-face interaction network (Research lab)

| Team | Edge-IDs | Detections |
| --- | --- | --- |
| Team_5 | 14-18 | 3,305 |
| Team_5 | 16-18 | 1,270 |
| Team_5 | 15-21 | 1,043 |
| Team_5 | 13-14 | 897 |
| Team_5 | 16-20 | 606 |
| Team_5 | 12-15 | 586 |
| Team_5 | 12-16 | 544 |
| Team_5 | 17-19 | 424 |
| Team_5 | 17-18 | 349 |
| Team_5 | 16-21 | 348 |


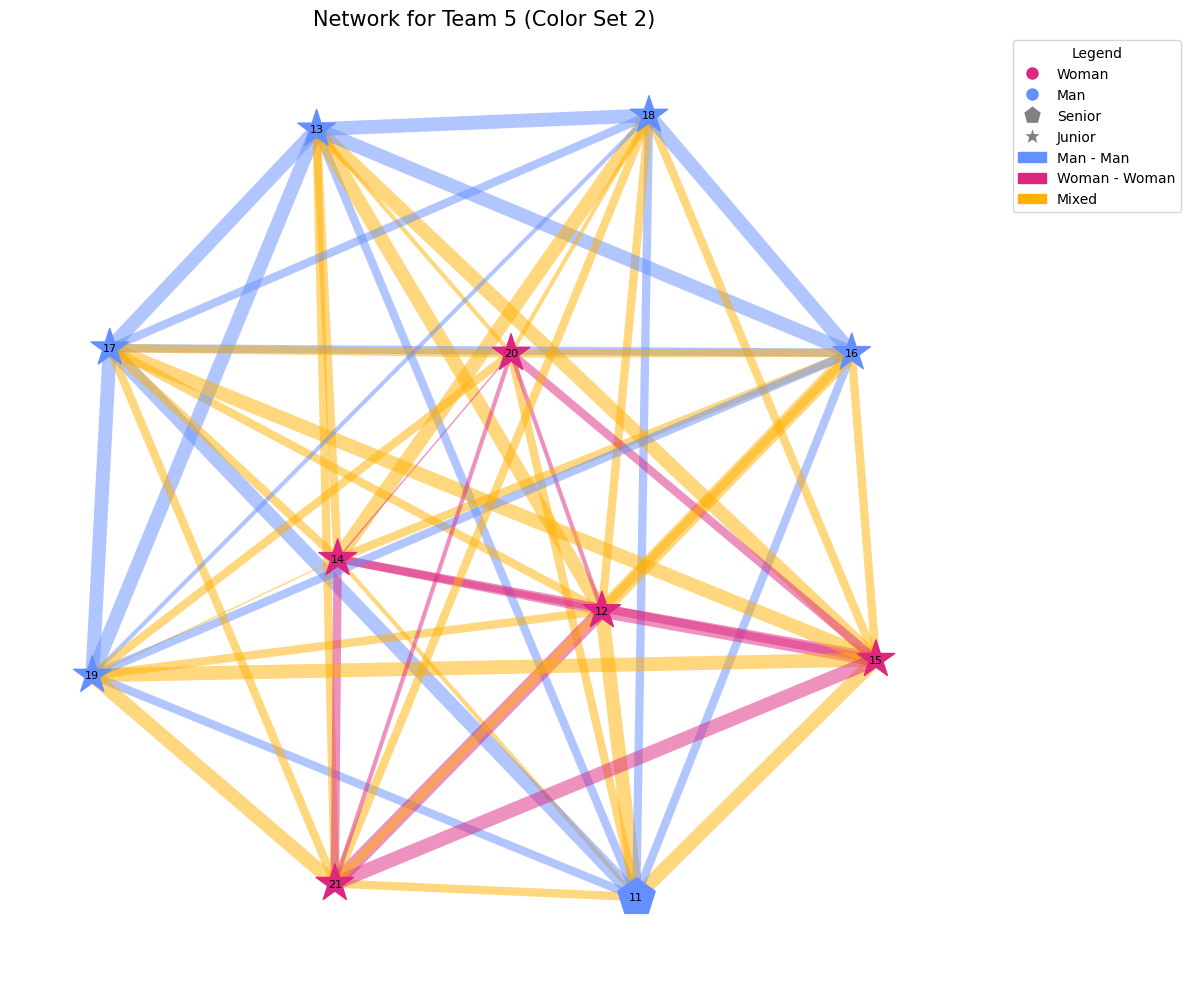


#### Team 6 face-to-face interaction network (University)

| Team | Edge-IDs | Detections |
| --- | --- | --- |
| Team_6 | 42-46 | 2,006 |
| Team_6 | 42-43 | 627 |
| Team_6 | 43-44 | 384 |
| Team_6 | 41-44 | 170 |
| Team_6 | 43-46 | 67 |
| Team_6 | 41-46 | 39 |
| Team_6 | 41-43 | 37 |
| Team_6 | 39-42 | 20 |
| Team_6 | 41-47 | 9 |
| Team_6 | 43-47 | 9 |


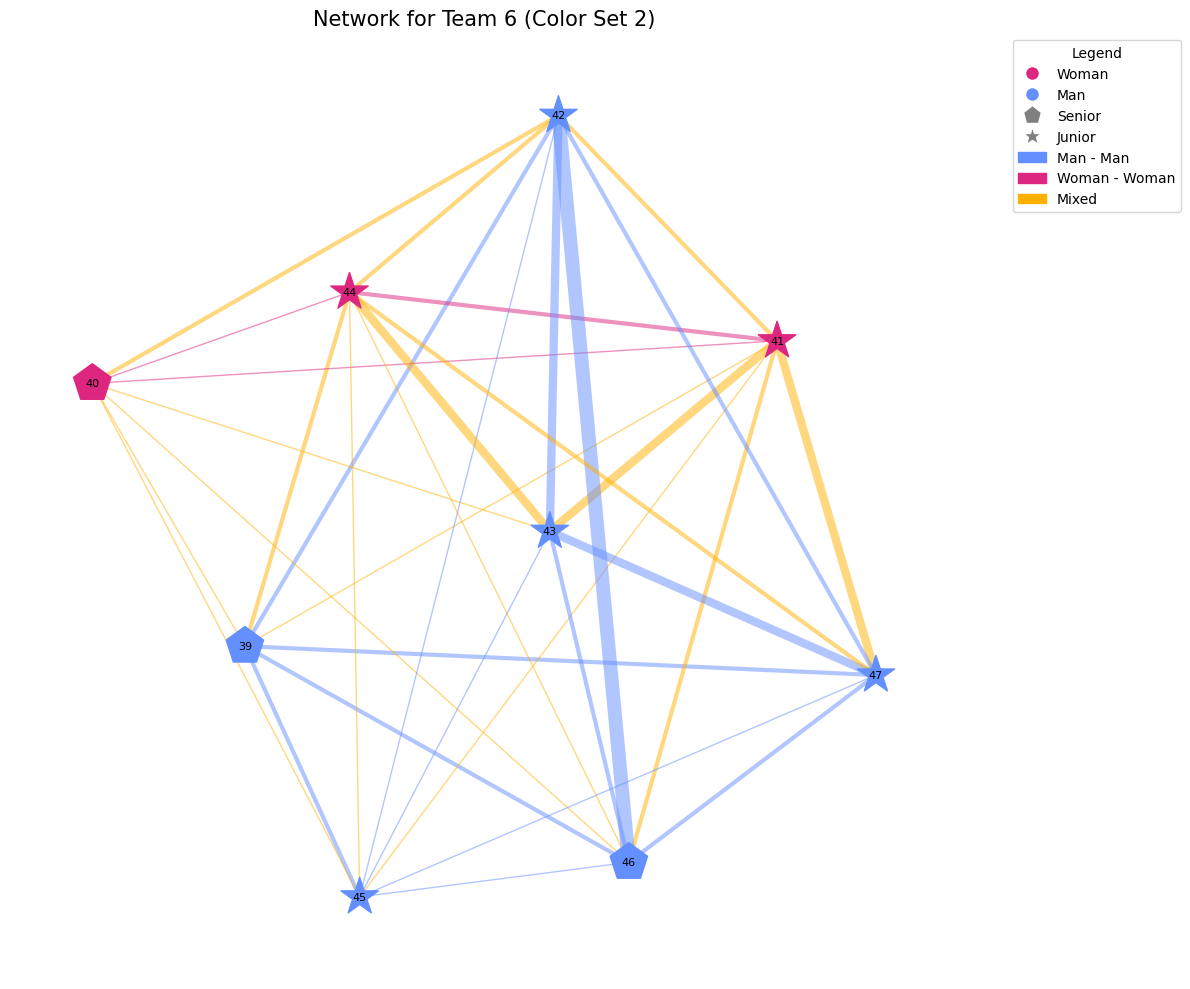


#### Team 7a face-to-face interaction network (Private company)

| Team | Edge-IDs | Detections |
| --- | --- | --- |
| Team_7A | 61-63 | 2,236 |
| Team_7A | 58-63 | 1,814 |
| Team_7A | 59-60 | 1,188 |
| Team_7A | 57-58 | 972 |
| Team_7A | 57-61 | 663 |
| Team_7A | 60-61 | 586 |
| Team_7A | 58-61 | 158 |
| Team_7A | 60-62 | 58 |
| Team_7A | 57-59 | 31 |
| Team_7A | 62-63 | 30 |


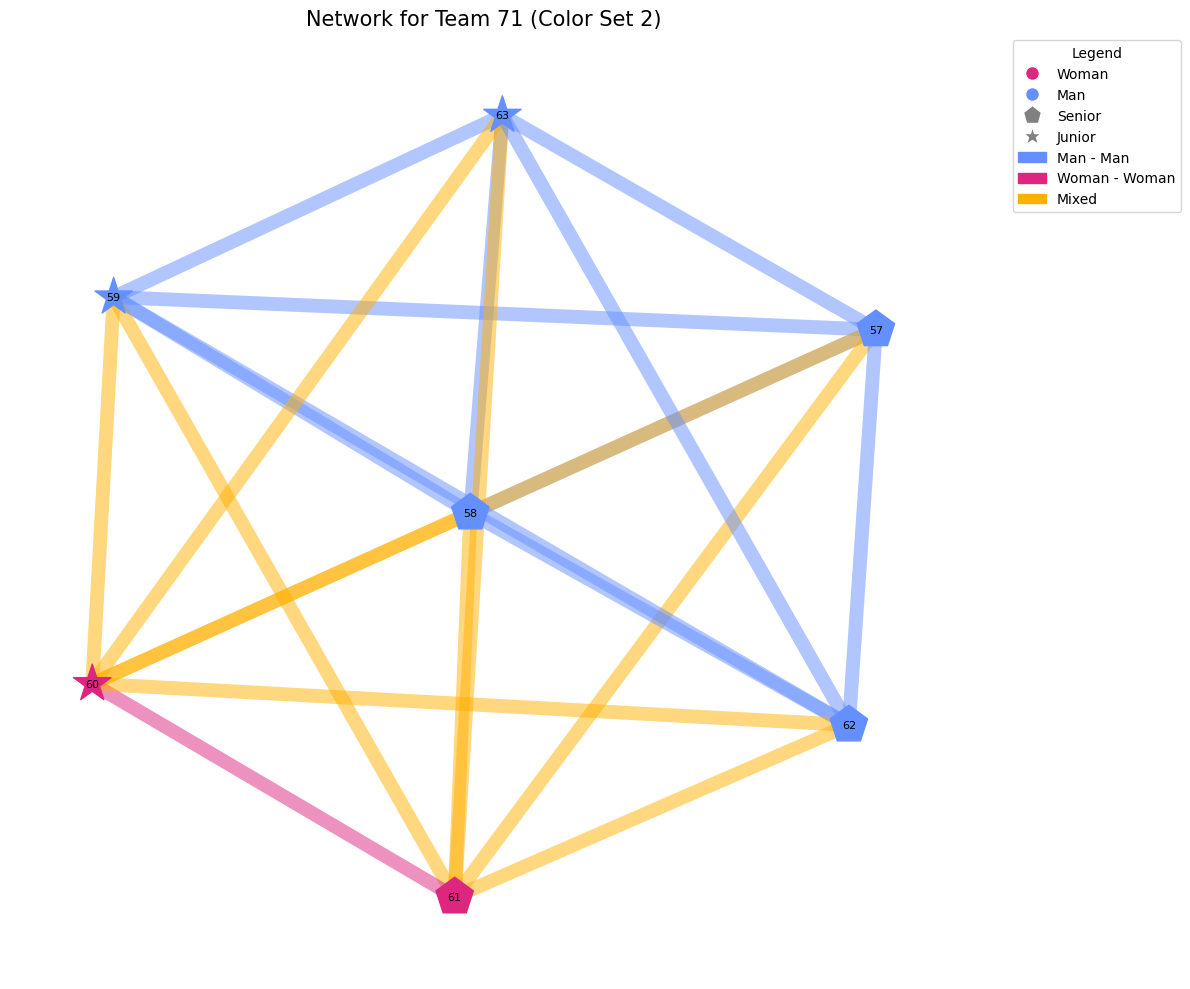


#### Team 7b face-to-face interaction network (Private company)

| Team | Edge-IDs | Detections |
| --- | --- | --- |
| Team_7B | 65-66 | 3,710 |
| Team_7B | 68-70 | 2,309 |
| Team_7B | 64-70 | 1,383 |
| Team_7B | 64-65 | 108 |
| Team_7B | 65-68 | 51 |
| Team_7B | 66-68 | 27 |
| Team_7B | 65-70 | 9 |


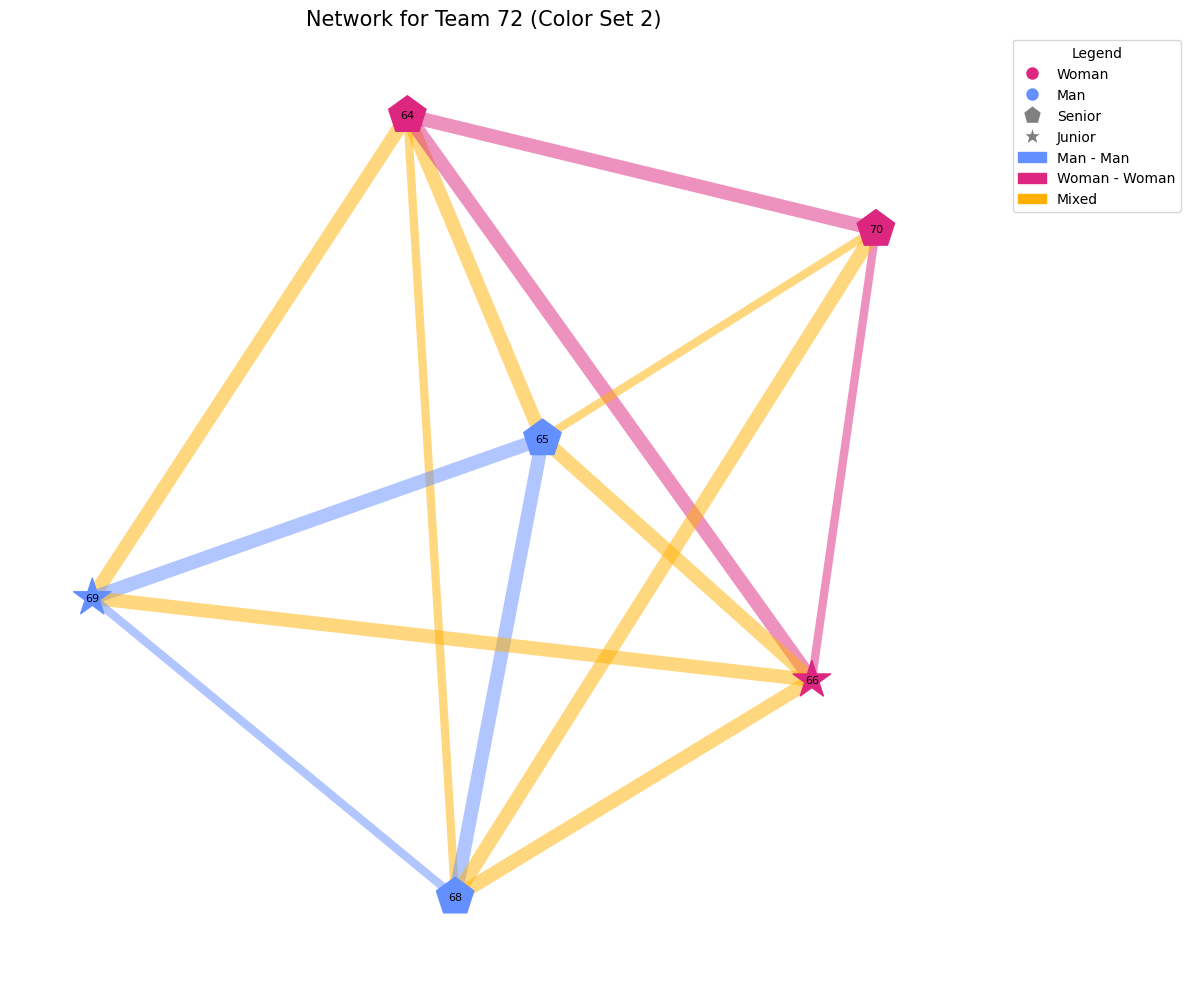


#### Team 8 face-to-face interaction network (Private company)

| Team | Edge-IDs | Detections |
| --- | --- | --- |
| Team_8 | 72-78 | 229 |
| Team_8 | 74-78 | 193 |
| Team_8 | 74-77 | 128 |
| Team_8 | 72-73 | 95 |
| Team_8 | 73-75 | 62 |
| Team_8 | 77-78 | 51 |
| Team_8 | 72-75 | 46 |
| Team_8 | 72-77 | 41 |
| Team_8 | 76-78 | 29 |
| Team_8 | 75-78 | 28 |


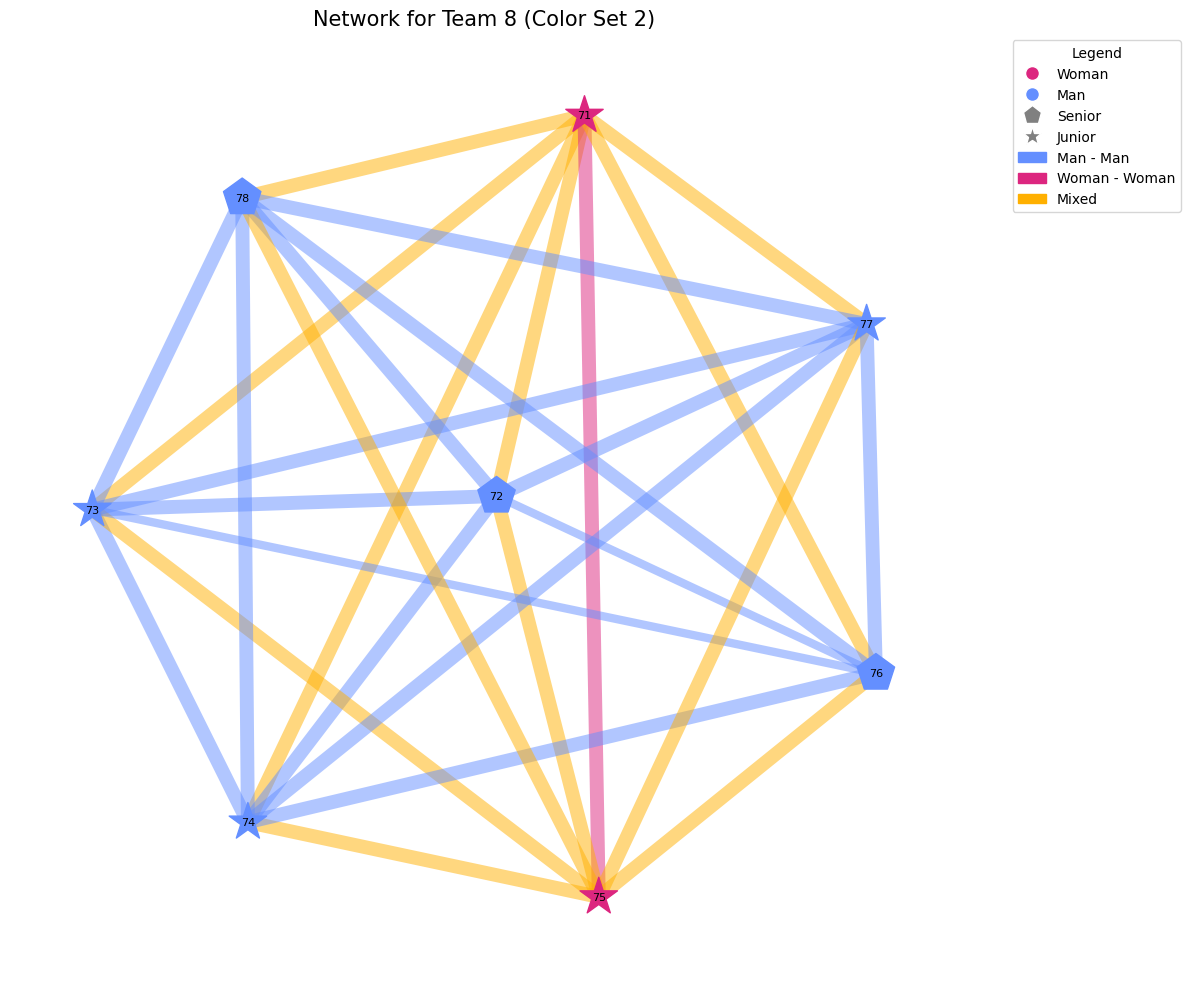


#### Team 9 face-to-face interaction network (Research lab)

| Team | Edge-IDs | Detections |
| --- | --- | --- |
| Team_9 | 102-92 | 1,247 |
| Team_9 | 88-94 | 966 |
| Team_9 | 91-94 | 959 |
| Team_9 | 100-92 | 676 |
| Team_9 | 100-101 | 622 |
| Team_9 | 101-92 | 425 |
| Team_9 | 91-92 | 305 |
| Team_9 | 81-84 | 253 |
| Team_9 | 88-91 | 189 |
| Team_9 | 101-91 | 166 |


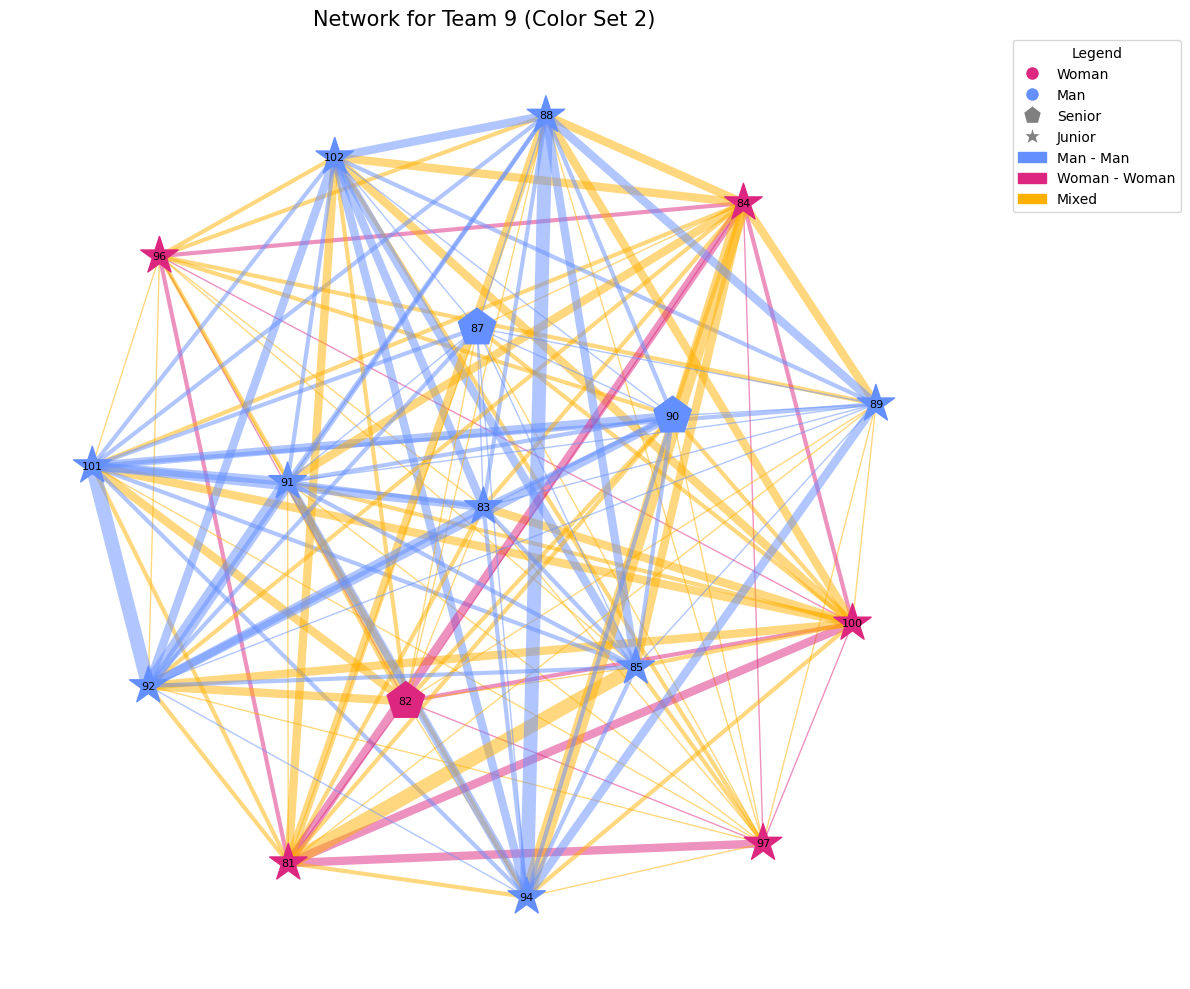


#### Team 10 face-to-face interaction network (Research lab)

| Team | Edge-IDs | Detections |
| --- | --- | --- |
| Team_10 | 107-108 | 366 |
| Team_10 | 104-110 | 347 |
| Team_10 | 103-106 | 327 |
| Team_10 | 105-107 | 275 |
| Team_10 | 103-108 | 176 |
| Team_10 | 103-107 | 117 |
| Team_10 | 103-110 | 76 |
| Team_10 | 104-105 | 75 |
| Team_10 | 108-109 | 15 |
| Team_10 | 104-108 | 14 |


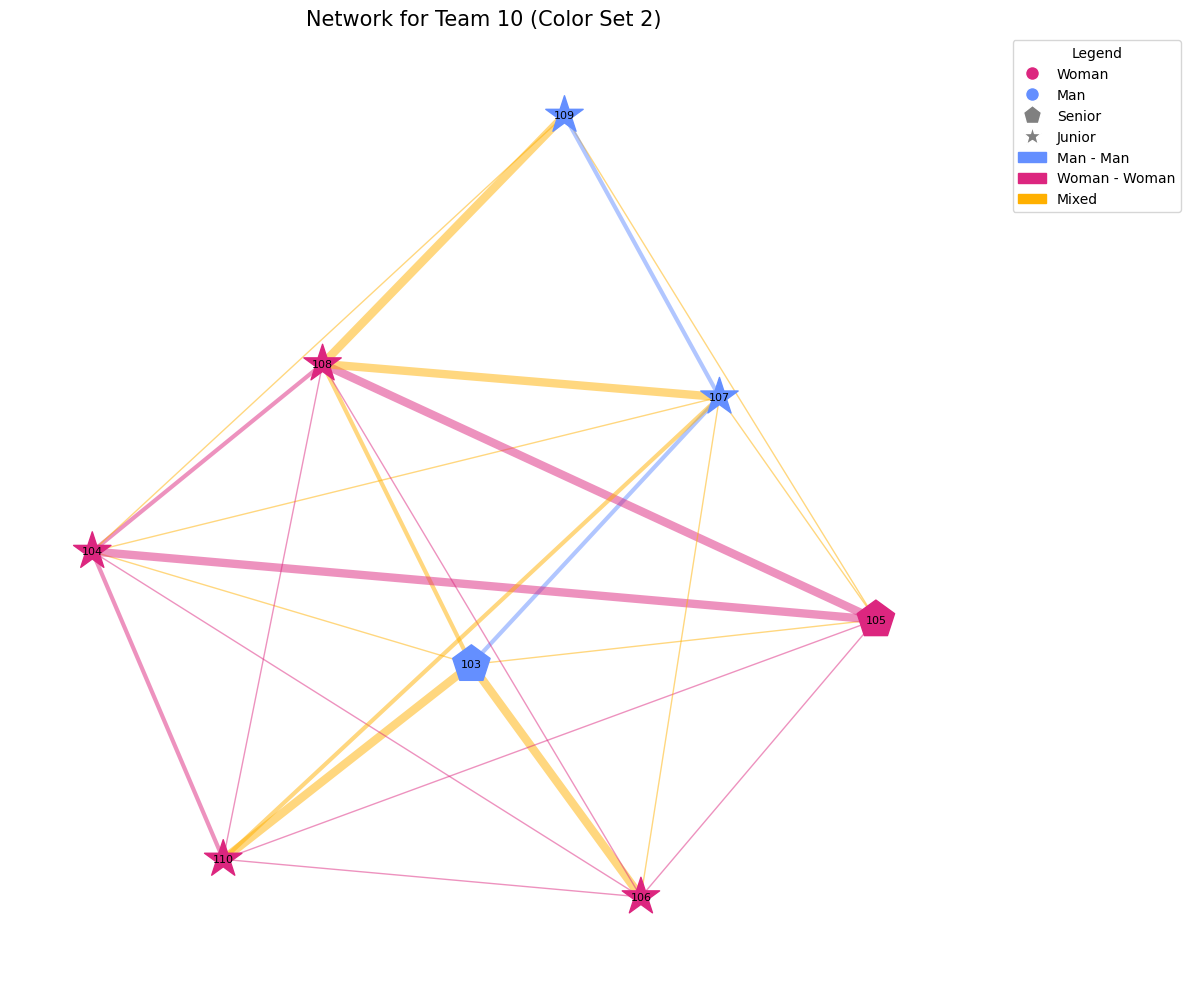

Supplement: S1 File — (DOCX) [file pone.0349195.s001.docx]
