# Supplementary material for: Gender, status, and team interaction: A microdynamic exploration of wearable sensor data across 11 research groups
Source: PLoS One. 2026 May 15;21(5):e0349195. doi: 10.1371/journal.pone.0349195 (PMC13178976; doi:10.1371/journal.pone.0349195)
Supplement: S2 File — (DOCX) [file pone.0349195.s002.docx]

**S2 – Supplementary Tables – DyNAM Model Statistics**

Table S1 Posterior summaries for DyNAM parameters across organisational contexts

| **Effect** | **Context** | **β** | **95% CI** | **pd** |
| --- | --- | --- | --- | --- |
| ***Gender homophily*** | | | | |
| Men dyad (β_4_) | University (*uni*) | 0.25 | [ -0.12, 0.55] | 0.90 |
|  | Research Lab (*lab*) | -0.21 | [ -0.45, 0.03] | 0.96 |
|  | Private Company (*bus*) | -0.80 | [ -1.28, -0.34] | 1.00 |
| Women dyad (β_5_) | University (*uni*) | -0.35 | [ -0.66, -0.06] | 0.99 |
|  | Research Lab (*lab*) | -0.54 | [ -0.75, -0.33] | 1.00 |
|  | Private Company (*bus*) | 0.17 | [ -1.18, 1.21] | 0.61 |
| ***Relationship effects*** | | | | |
| Friendship tie (β_6_) | University (*uni*) | 0.71 | [ 0.27, 1.18] | 1.00 |
|  | Research Lab (*lab*) | 0.00 | [ -0.19, 0.19] | 0.51 |
|  | Private Company (*bus*) | 1.01 | [ 0.47, 1.54] | 1.00 |
| Advice tie (β_7_) | University (*uni*) | 0.20 | [ -0.06, 0.46] | 0.93 |
|  | Research Lab (*lab*) | 0.30 | [ 0.16, 0.43] | 1.00 |
|  | Private Company (*bus*) | 1.52 | [ 0.95, 2.15] | 1.00 |
| ***Status effects*** | | | | |
| Junior (β_9_) | University (*uni*) | -0.19 | [ -0.45, 0.07] | 0.93 |
|  | Research Lab (*lab*) | -0.13 | [ -0.27, 0.02] | 0.96 |
|  | Private Company (*bus*) | 0.41 | [ -0.28, 1.06] | 0.88 |
| Senior (β_10_) | University (*uni*) | 0.19 | [ -0.06, 0.44] | 0.93 |
|  | Research Lab (*lab*) | -0.05 | [ -0.23, 0.12] | 0.72 |
|  | Private Company (*bus*) | -0.50 | [ -0.90, -0.12] | 1.00 |
| ***Gender x Relationship*** | | | | |
| Men dyad x  Advice (β_11_) | University (*uni*) | -0.02 | [ -0.57, 0.58] | 0.51 |
|  | Research Lab (*lab*) | 0.03 | [ -0.21, 0.26] | 0.58 |
|  | Private Company (*bus*) | -0.59 | [ -1.40, 0.17] | 0.93 |
| Women dyad x  Advice (β_12_) | University (*uni*) | 0.19 | [ -0.43, 0.85] | 0.73 |
|  | Research Lab (*lab*) | 0.39 | [ 0.17, 0.61] | 1.00 |
|  | Private Company (*bus*) | -1.11 | [ -2.38, 0.42] | 0.93 |
| Men dyad x  Friendship (β_13_) | University (*uni*) | -0.05 | [ -0.69, 0.63] | 0.56 |
|  | Research Lab (*lab*) | 0.40 | [ 0.13, 0.67] | 1.00 |
|  | Private Company (*bus*) | -0.23 | [ -1.12, 0.68] | 0.69 |
| Women dyad x  Friendship (β_14_) | University (*uni*) | -0.02 | [ -1.19, 1.28] | 0.51 |
|  | Research Lab (*lab*) | 0.71 | [ 0.42, 1.00] | 1.00 |
|  | Private Company (*bus*) | -4.78 | [-15.56, 0.70] | 0.94 |
| ***Gender x status*** | | | | |
| Men dyad x  Senior (β_15_) | University (*uni*) | -0.09 | [ -0.47, 0.31] | 0.65 |
|  | Research Lab (*lab*) | 0.36 | [ 0.03, 0.68] | 0.98 |
|  | Private Company (*bus*) | 0.41 | [ -0.10, 0.94] | 0.94 |
| Women dyad x  Senior (β_16_) | University (*uni*) | -0.05 | [ -0.88, 0.60] | 0.56 |
|  | Research Lab (*lab*) | 0.22 | [ -0.02, 0.45] | 0.96 |
|  | Private Company (*bus*) | 1.43 | [ 0.65, 2.30] | 1.00 |
| Men dyad x  Junior (β_17_) | University (*uni*) | -0.07 | [ -0.43, 0.28] | 0.65 |
|  | Research Lab (*lab*) | 0.36 | [ 0.17, 0.56] | 1.00 |
|  | Private Company (*bus*) | 0.01 | [ -7.64, 7.70] | 0.50 |
| Women dyad x  Junior (β_18_) | University (*uni*) | 0.33 | [ -0.16, 0.76] | 0.91 |
|  | Research Lab (*lab*) | 0.46 | [ 0.28, 0.63] | 1.00 |
|  | Private Company (*bus*) | -0.00 | [ -8.10, 8.08] | 0.50 |
| ***Endogenous effects*** | | | | |
| Inertia (γ_1_) | University (*uni*) | -0.52 | [ -0.92, -0.15] | 0.99 |
|  | Research Lab (*lab*) | -0.37 | [ -0.79, 0.05] | 0.96 |
|  | Private Company (*bus*) | -0.65 | [ -1.22, -0.08] | 0.99 |
| Degree (β_2_) | University (*uni*) | 1.30 | [ 0.84, 1.76] | 1.00 |
|  | Research Lab (*lab*) | 1.05 | [ 0.73, 1.37] | 1.00 |
|  | Private Company (*bus*) | 0.49 | [ -0.40, 1.37] | 0.86 |
| Recency (β_3_) | University (*uni*) | 1.41 | [ 1.24, 1.58] | 1.00 |
|  | Research Lab (*lab*) | 1.10 | [ 0.99, 1.20] | 1.00 |
|  | Private Company (*bus*) | 1.99 | [ 1.66, 2.32] | 1.00 |
| ***Alternative explanations*** | | | | |
| Colocation (β_8_) | University (*uni*) | 0.15 | [ -0.01, 0.32] | 0.97 |
|  | Research Lab (*lab*) | 0.09 | [ -0.02, 0.20] | 0.95 |
|  | Private Company (*bus*) | -0.35 | [ -0.96, 0.28] | 0.86 |
| Homophily age (β_19_) | University (*uni*) | -0.10 | [ -0.27, 0.07] | 0.87 |
|  | Research Lab (*lab*) | 0.10 | [ 0.01, 0.19] | 0.99 |
|  | Private Company (*bus*) | -0.02 | [ -0.23, 0.21] | 0.57 |
| Homophily tenure (β_20_) | University (*uni*) | -0.07 | [ -0.20, 0.05] | 0.88 |
|  | Research Lab (*lab*) | 0.05 | [ -0.05, 0.16] | 0.85 |
|  | Private Company (*bus*) | -0.22 | [ -0.54, 0.09] | 0.91 |
| Notes: 95% CI, the equal-tailed 95% credible interval; pd, probability of direction | | | | |

Table S2 Posterior summaries for pairwise differences in beta parameters across organisational contexts

| **Effect** | **Comparison** | **Δβ** | **95% CI** | **pd** |
| --- | --- | --- | --- | --- |
| ***Gender homophily*** | | | | |
| Men dyad (Δβ_4_) | *lab - uni* | -0.45 | [ -0.85, -0.02] | 0.98 |
|  | *bus - uni* | -1.05 | [ -1.63, -0.45] | 1.00 |
|  | *bus - lab* | -0.59 | [ -1.13, -0.08] | 0.99 |
| Women dyad (Δβ_5_) | *lab - uni* | -0.19 | [ -0.55, 0.18] | 0.85 |
|  | *bus - uni* | 0.52 | [ -0.86, 1.61] | 0.79 |
|  | *bus - lab* | 0.71 | [ -0.65, 1.77] | 0.86 |
| ***Relationship effects*** | | | | |
| Friendship tie (Δβ_6_) | *lab - uni* | -0.71 | [ -1.21, -0.24] | 1.00 |
|  | *bus - uni* | 0.29 | [ -0.42, 0.98] | 0.79 |
|  | *bus - lab* | 1.01 | [ 0.44, 1.57] | 1.00 |
| Advice tie (Δβ_7_) | *lab - uni* | 0.10 | [ -0.20, 0.40] | 0.74 |
|  | *bus - uni* | 1.32 | [ 0.68, 1.99] | 1.00 |
|  | *bus - lab* | 1.22 | [ 0.63, 1.87] | 1.00 |
| ***Status effects*** | | | | |
| Junior (Δβ_9_) | *lab - uni* | 0.07 | [ -0.23, 0.36] | 0.67 |
|  | *bus - uni* | 0.60 | [ -0.14, 1.30] | 0.95 |
|  | *bus - lab* | 0.53 | [ -0.17, 1.20] | 0.93 |
| Senior (Δβ_10_) | *lab - uni* | -0.24 | [ -0.55, 0.06] | 0.94 |
|  | *bus - uni* | -0.69 | [ -1.16, -0.24] | 1.00 |
|  | *bus - lab* | -0.45 | [ -0.88, -0.03] | 0.98 |
| ***Gender x Relationship*** | | | | |
| Men dyad x  Advice (Δβ_11_) | *lab - uni* | 0.04 | [ -0.61, 0.65] | 0.53 |
|  | *bus - uni* | -0.58 | [ -1.65, 0.40] | 0.87 |
|  | *bus - lab* | -0.62 | [ -1.45, 0.18] | 0.93 |
| Women dyad x  Advice (Δβ_12_) | *lab - uni* | 0.20 | [ -0.49, 0.86] | 0.71 |
|  | *bus - uni* | -1.31 | [ -2.73, 0.34] | 0.94 |
|  | *bus - lab* | -1.51 | [ -2.79, 0.04] | 0.97 |
| Men dyad x  Friendship (Δβ_13_) | *lab - uni* | 0.45 | [ -0.28, 1.15] | 0.89 |
|  | *bus - uni* | -0.17 | [ -1.30, 0.94] | 0.62 |
|  | *bus - lab* | -0.63 | [ -1.56, 0.32] | 0.90 |
| Women dyad x  Friendship (Δβ_14_) | *lab - uni* | 0.73 | [ -0.61, 1.93] | 0.86 |
|  | *bus - uni* | -4.81 | [-15.58, 0.85] | 0.94 |
|  | *bus - lab* | -5.49 | [-16.26, -0.01] | 0.98 |
| ***Gender x status*** | | | | |
| Men dyad x  Senior (Δβ_15_) | *lab - uni* | 0.45 | [ -0.07, 0.95] | 0.95 |
|  | *bus - uni* | 0.50 | [ -0.15, 1.14] | 0.93 |
|  | *bus - lab* | 0.05 | [ -0.56, 0.67] | 0.56 |
| Women dyad x  Senior (Δβ_16_) | *lab - uni* | 0.27 | [ -0.43, 1.13] | 0.77 |
|  | *bus - uni* | 1.50 | [ 0.45, 2.68] | 1.00 |
|  | *bus - lab* | 1.21 | [ 0.39, 2.12] | 1.00 |
| Men dyad x  Junior (Δβ_17_) | *lab - uni* | 0.43 | [ 0.03, 0.84] | 0.98 |
|  | *bus - uni* | 0.08 | [ -7.57, 7.77] | 0.51 |
|  | *bus - lab* | -0.35 | [ -8.00, 7.34] | 0.54 |
| Women dyad x  Junior (Δβ_18_) | *lab - uni* | 0.13 | [ -0.34, 0.64] | 0.69 |
|  | *bus - uni* | -0.33 | [ -8.44, 7.79] | 0.53 |
|  | *bus - lab* | -0.45 | [ -8.56, 7.63] | 0.54 |
| ***Endogenous effects*** | | | | |
| Inertia (Δγ_1_) | *lab - uni* | 0.14 | [ -0.40, 0.74] | 0.71 |
|  | *bus - uni* | -0.13 | [ -0.80, 0.58] | 0.66 |
|  | *bus - lab* | -0.28 | [ -0.98, 0.43] | 0.80 |
| Degree (Δβ_2_) | *lab - uni* | -0.24 | [ -0.80, 0.31] | 0.81 |
|  | *bus - uni* | -0.81 | [ -1.81, 0.18] | 0.95 |
|  | *bus - lab* | -0.56 | [ -1.51, 0.37] | 0.88 |
| Recency (Δβ_3_) | *lab - uni* | -0.31 | [ -0.51, -0.12] | 1.00 |
|  | *bus - uni* | 0.57 | [ 0.21, 0.95] | 1.00 |
|  | *bus - lab* | 0.89 | [ 0.55, 1.24] | 1.00 |
| ***Alternative explanations*** | | | | |
| Colocation (Δβ_8_) | *lab - uni* | -0.06 | [ -0.26, 0.13] | 0.74 |
|  | *bus - uni* | -0.51 | [ -1.14, 0.15] | 0.94 |
|  | *bus - lab* | -0.44 | [ -1.06, 0.20] | 0.91 |
| Homophily age (Δβ_19_) | *lab - uni* | 0.20 | [ 0.01, 0.39] | 0.98 |
|  | *bus - uni* | 0.08 | [ -0.19, 0.37] | 0.71 |
|  | *bus - lab* | -0.12 | [ -0.35, 0.13] | 0.83 |
| Homophily tenure (Δβ_20_) | *lab - uni* | 0.13 | [ -0.03, 0.29] | 0.94 |
|  | *bus - uni* | -0.15 | [ -0.48, 0.19] | 0.80 |
|  | *bus - lab* | -0.27 | [ -0.61, 0.05] | 0.95 |
| Notes: Δβ, difference in β parameters between organisational contexts; | | | | |
| 95% CI, the equal-tailed 95% credible interval; pd, probability of direction; | | | | |
| uni, university; lab, research lab; bus, private company | | | | |

Table S3 Posterior summaries of probability ratios for gender homophily by relationship tie and organisational context

| **Gender Contrast** | **Context** | **PR** | **95% CI** | **pd** |
| --- | --- | --- | --- | --- |
| ***Reference*** | | | | |
| Women dyad / Mix gender dyad | University | 0.50 | [0.27, 0.89] | 0.99 |
|  | Research Lab | 0.34 | [0.22, 0.51] | 1.00 |
|  | Private Company | 1.40 | [0.09, 11.2] | 0.61 |
| Men dyad / Mix gender dyad | University | 1.66 | [0.79, 3.02] | 0.90 |
|  | Research Lab | 0.66 | [0.41, 1.06] | 0.96 |
|  | Private Company | 0.20 | [0.08, 0.50] | 1.00 |
| Women dyad / Men dyad | University | 0.30 | [0.13, 0.77] | 0.99 |
|  | Research Lab | 0.51 | [0.28, 0.95] | 0.98 |
|  | Private Company | 6.88 | [0.39, 72.5] | 0.91 |
| ***Advice*** | | | | |
| Women dyad / Mix gender dyad | University | 1.08 | [0.11, 11.4] | 0.53 |
|  | Research Lab | 1.63 | [0.82, 3.22] | 0.92 |
|  | Private Company | 0.02 | [0.00, 0.89] | 0.98 |
| Men dyad / Mix gender dyad | University | 1.59 | [0.24, 10.1] | 0.64 |
|  | Research Lab | 0.73 | [0.34, 1.54] | 0.79 |
|  | Private Company | 0.02 | [0.00, 0.29] | 1.00 |
| Women dyad / Men dyad | University | 0.46 | [0.12, 1.73] | 0.87 |
|  | Research Lab | 1.07 | [0.64, 1.79] | 0.60 |
|  | Private Company | 2.50 | [0.42, 12.9] | 0.85 |
| ***Friendship*** | | | | |
| Women dyad / Mix gender dyad | University | 0.48 | [0.04, 6.46] | 0.71 |
|  | Research Lab | 1.40 | [0.77, 2.52] | 0.87 |
|  | Private Company | 0.00 | [0.00, 3.43] | 0.94 |
| Men dyad / Mix gender dyad | University | 1.45 | [0.37, 6.44] | 0.69 |
|  | Research Lab | 1.47 | [0.89, 2.42] | 0.93 |
|  | Private Company | 0.13 | [0.02, 1.09] | 0.97 |
| Women dyad / Men dyad | University | 0.33 | [0.02, 6.16] | 0.77 |
|  | Research Lab | 0.95 | [0.45, 2.00] | 0.55 |
|  | Private Company | 0.00 | [0.00, 32.9] | 0.86 |
| ***Main Effects*** | | | | |
| Advice / No advice | University | 1.49 | [0.88, 2.51] | 0.93 |
|  | Research Lab | 1.81 | [1.37, 2.38] | 1.00 |
|  | Private Company | 20.7 | [6.64, 73.2] | 1.00 |
| Friend / No friend | University | 4.17 | [1.72, 10.6] | 1.00 |
|  | Research Lab | 1.00 | [0.69, 1.45] | 0.51 |
|  | Private Company | 7.50 | [2.56, 21.7] | 1.00 |
| Notes: PR, probability ratio computed as exp(2 × linear predictor); | | | | |
| 95% CI, the equal-tailed 95% credible interval; pd, probability of direction | | | | |

Table S4 Posterior summaries of probability ratios for gender homophily by seniority status and organisational context

| **Gender Contrast** | **Context** | **PR** | **95% CI** | **pd** |
| --- | --- | --- | --- | --- |
| ***Reference*** | | | | |
| Women dyad / Mix gender dyad | University | 0.50 | [0.27, 0.89] | 0.99 |
|  | ResearchLab | 0.34 | [0.22, 0.51] | 1.00 |
|  | PrivateCompany | 1.40 | [0.09, 11.2] | 0.61 |
| Men dyad / Mix gender dyad | University | 1.66 | [0.79, 3.02] | 0.90 |
|  | ResearchLab | 0.66 | [0.41, 1.06] | 0.96 |
|  | PrivateCompany | 0.20 | [0.08, 0.50] | 1.00 |
| Women dyad / Men dyad | University | 0.30 | [0.13, 0.77] | 0.99 |
|  | ResearchLab | 0.51 | [0.28, 0.95] | 0.98 |
|  | PrivateCompany | 6.88 | [0.39, 72.5] | 0.91 |
| ***Junior*** | | | | |
| Women dyad / Mix gender dyad | University | 0.98 | [0.32, 2.22] | 0.52 |
|  | ResearchLab | 0.84 | [0.57, 1.23] | 0.81 |
|  | PrivateCompany | 1.30 | [0.00, 1.6e+07] | 0.51 |
| Men dyad / Mix gender dyad | University | 1.43 | [0.52, 3.56] | 0.75 |
|  | ResearchLab | 1.36 | [0.92, 2.03] | 0.94 |
|  | PrivateCompany | 0.20 | [0.00, 1e+06] | 0.58 |
| Women dyad / Men dyad | University | 0.68 | [0.18, 2.35] | 0.72 |
|  | ResearchLab | 0.62 | [0.36, 1.04] | 0.96 |
|  | PrivateCompany | 6.37 | [0.00, 3.5e+10] | 0.56 |
| ***Senior*** | | | | |
| Women dyad / Mix gender dyad | University | 0.44 | [0.09, 1.54] | 0.89 |
|  | ResearchLab | 0.52 | [0.30, 0.90] | 0.99 |
|  | PrivateCompany | 25.0 | [1.58, 200] | 0.99 |
| Men dyad / Mix gender dyad | University | 1.39 | [0.43, 4.21] | 0.66 |
|  | ResearchLab | 1.37 | [0.68, 2.68] | 0.82 |
|  | PrivateCompany | 0.46 | [0.18, 1.17] | 0.95 |
| Women dyad / Men dyad | University | 0.31 | [0.04, 1.88] | 0.89 |
|  | ResearchLab | 0.38 | [0.17, 0.88] | 0.99 |
|  | PrivateCompany | 54.3 | [3.17, 480] | 0.99 |
| ***Main Effects*** | | | | |
| Junior-Junior / Junior-Senior | University | 0.68 | [0.41, 1.15] | 0.93 |
|  | ResearchLab | 0.78 | [0.58, 1.04] | 0.96 |
|  | PrivateCompany | 2.27 | [0.57, 8.35] | 0.88 |
| Senior-Senior / Junio-Junior | University | 2.16 | [1.04, 4.47] | 0.98 |
|  | ResearchLab | 1.16 | [0.74, 1.81] | 0.74 |
|  | PrivateCompany | 0.16 | [0.03, 0.78] | 0.99 |
| Senior-Senior / Junior-Senior | University | 1.46 | [0.89, 2.42] | 0.93 |
|  | ResearchLab | 0.90 | [0.64, 1.27] | 0.72 |
|  | PrivateCompany | 0.37 | [0.17, 0.78] | 1.00 |
| Notes: PR, probability ratio computed as exp(2 × linear predictor); | | | | |
| 95% CI, the equal-tailed 95% credible interval; pd, probability of direction | | | | |

Table S5 Posterior summaries for team-specific random effects

| **Team ID** | **γ** | **95% CI** | **pd** |
| --- | --- | --- | --- |
| ***University*** | | | |
| 9 | 0.30 | [-0.03, 0.71] | 0.96 |
| 1 | 0.11 | [-0.29, 0.63] | 0.72 |
| 6 | 0.10 | [-0.30, 0.59] | 0.70 |
| 10 | -0.12 | [-0.57, 0.28] | 0.73 |
| 3 | -0.43 | [-0.99, -0.01] | 0.98 |
| ***Research Lab*** | | | |
| 5 | 0.04 | [-0.38, 0.47] | 0.59 |
| 4 | -0.02 | [-0.47, 0.41] | 0.55 |
| 2 | -0.02 | [-0.46, 0.40] | 0.56 |
| ***Private Company*** | | | |
| 8 | 0.10 | [-0.35, 0.65] | 0.69 |
| 7A | -0.04 | [-0.54, 0.44] | 0.57 |
| 7B | -0.08 | [-0.67, 0.40] | 0.65 |
| Notes: γ, team-specific centered random effects; | | | |
| 95% CI, the equal-tailed 95% credible interval; pd, probability of direction. | | | |

Table S6 Posterior summaries for the standard deviation of team-specific random effects

| **Parameter** | **σ** | **95% CI** | **pd** |
| --- | --- | --- | --- |
| Random effects SD | 0.14 | [0.05, 0.32] | 1.00 |
| Notes: σ, the standard deviation of team-specific random effects; | | | |
| 95% CI, the equal-tailed 95% credible interval; pd, probability of direction | | | |
