# Supplementary material for: Gender, status, and team interaction: A microdynamic exploration of wearable sensor data across 11 research groups
Source: PLoS One. 2026 May 15;21(5):e0349195. doi: 10.1371/journal.pone.0349195 (PMC13178976; doi:10.1371/journal.pone.0349195)
Supplement: Attachment — Submitted filename: Review of Manuscript PONE.pdf [file pone.0349195.s003.pdf]

Review of Manuscript PONE-D-25-13840

"*Gender, Status, and Team Interaction: A Microdynamic Exploration of Wearable Sensor Data Across 11 Research Groups"*

This manuscript presents a methodologically interesting case study applying a hierarchical Dynamic Actor Network Model (DyNAM) to wearable sensor data capturing face-to-face interactions within R&D teams. The empirical setting of exploring team dynamics using such granular, real-world data is relevant and aligns well with PLOS ONE's interest in methodologically rigorous research. The application of DyNAM to this specific context also appears correct and represents a valuable extension of this method to new empirical settings, particularly within organizational studies.

Several areas require significant attention to strengthen the theoretical framing, methodological reporting, data justification, and overall presentation for publication in PLOS ONE.

1. **Literature Review and Theoretical Framing**
    - While the paper correctly identifies the shift towards a dynamic understanding of team processes and appropriately uses a DyNAM, the abstract and likely the full paper could benefit from a more comprehensive engagement with the broader literature on using social network models, particularly event-based models like Relational Event Models (REMs) and actor-based models, to study organizational settings. Key works by researchers such as Pilny, Lendeers, and Contractor – just to name a few – have significantly contributed to this area and should be referenced to properly contextualize the study within the existing field of organizational studies using dynamic network models.
    - It is also recommended that the authors include a reference to Bianchi et al.'s (2024) recent review on Relational Event Modeling. This review provides an overview of recent methodological and empirical developments in event-based network analysis, which is directly relevant to the paper's chosen method. Citing this review will also provide readers with valuable guidance on the state of the art in this domain.
    - The paper presents a substantial number of hypotheses (10, as noted). While exploring multiple facets of interaction is valuable, presenting this many distinct hypotheses in a single paper can sometimes dilute the core theoretical argument. The authors should review and potentially consolidate the hypotheses, ensuring each is clearly derived from a well-articulated theoretical argument and connected explicitly to the existing literature on gender, status, and team interaction dynamics.
2. **Dataset and Sampling Approach**
    - A significant concern arises from the heterogeneity of the dataset used in the analysis. Combining data from R&D teams collected in different years (2016/2017 vs. 2018), across different countries (UK and Spain), from varied organizational sectors (private vs. public), different environments (university, research labs, private company), and diverse scientific fields (biomedical engineering, construction, nanoscience, energy engineering) raises questions about the comparability of these groups.
    - Specifically, the perception and influence of "status" can vary significantly across national cultures (UK vs. Spain), organizational types (university vs. private company), and professional fields. Aggregating data without clearly accounting for these potential sources of variation might obscure or misinterpret the actual dynamics at play.
    - The splitting of "Team 7" into sections A and B also requires clear justification. Is this split based on a meaningful organizational or interactional boundary within that group, or is it an artifact of data collection or analytical choices? The rationale for this split and how data from these two sections are treated in the hierarchical model should be explicitly explained.
3. **Methodological Reporting**
    - While the abstract mentions a hierarchical DyNAM, the full paper appears to lack a clear, explicit mathematical equation specifying the model used. For a complex statistical model like DyNAM, a formal equation is crucial for clarity, reproducibility, and for reviewers and readers to fully understand how the different effects (gender, status, homophily, team-level variations) are modeled.

The current approach of inferring the model specification from hypotheses and figures is insufficient.

- The term "microdynamic perspective" is used in the abstract and likely in the paper. While the intuitive meaning – dynamic interactions at the individual/dyadic level and their emergent patterns – is understandable, providing a more explicit definition grounded in the literature would be beneficial. Clarifying that this perspective emphasizes how higher-level network configurations or team processes emerge from the aggregation of local, time-varying interactions would enhance conceptual clarity.

4. **Writing and Formatting**
    - Attention to detail in writing and formatting is important for a professional publication. On page 9 ("status expectations...."), missing spaces around inline mathematical notation need to be corrected.
    - The use of contractions (e.g., "It's" on page 17) should be reviewed and made consistent with the overall writing style of the manuscript.

In conclusion, the paper presents a valuable application of dynamic network modeling to a relevant topic, and addressing the identified points is crucial to enhance its quality and impact.
