# Supplementary material for: Gender, status, and team interaction: A microdynamic exploration of wearable sensor data across 11 research groups
Source: PLoS One. 2026 May 15;21(5):e0349195. doi: 10.1371/journal.pone.0349195 (PMC13178976; doi:10.1371/journal.pone.0349195)
Supplement: Attachment — Submitted filename: PONE-D-25-13840_R1.pdf [file pone.0349195.s005.pdf]

Manuscript Number: PONE-D-25-13840R1

Gender, Status, and Team Interaction: A Microdynamic Exploration of Wearable Sensor Data Across 11 Research Groups

The authors submitted a revised manuscript analyzing gender, status, and team interaction. While the application of the Dynamic Actor Network Model (DyNAM) within a Bayesian framework is methodologically robust, the interpretation of the results remains flawed. The current analysis fails to exploit the advantages of the Bayesian apparatus.

The authors apply a strict frequentist heuristic to Bayesian results–rejecting any hypothesis where the 95% Credible Interval (CI) includes zero (or one for ratios). This binary approach discards the probabilistic information contained in the posterior distributions. A major revision is necessary to align the interpretation with the analytical framework used.

1. **Underutilization of the Bayesian Framework**

The authors state in the Methods: "we consider there to be strong statistical evidence for an effect if the 95% CI strictly excludes zero." This mimics a frequentist $p < 0.05$ test and negates the benefits of Bayesian inference. By relying on arbitrary cut-offs, the manuscript dismisses effects that may have a high probability of existence but high uncertainty.

For example, in the results for Hypothesis 3d (Junior Men), the text mentions a "probability of 96% that PR is above 1". In a Bayesian context, a 96% probability that an effect exists is substantial evidence. Yet the authors frequently revert to "rejecting" or labeling results "inconclusive" solely because the 95% interval touches the null value.

The authors must abandon the binary "significant/not significant" language. To fix this while retaining the Bayesian approach, I suggest the following:

- **Report the Probability of Direction (pd).** Calculate and report the percentage of the posterior distribution that shares the same sign as the median. This provides a direct probability statement (e.g., "there is a 93% probability that gender homophily is positive") which is more intuitive and accurate than a CI-based rejection.
- **Discuss Magnitude and Uncertainty.** Instead of simply accepting or rejecting hypotheses, discuss the *strength* of the evidence provided by the posterior. If 90% of the posterior mass is positive, this is worth discussing as a probable effect, even if the 95% CI includes zero.
- **Reformulate Conclusions.** Re-evaluate the "rejected" hypotheses (e.g., H2a, H3a) using these probabilistic metrics. It is likely that the data provides stronger support for your theoretical claims than the current binary interpretation suggests.

2. **Interpretation of "Contradictory" Contexts**

The manuscript characterizes differences between the University, Lab, and Private Company contexts as "contradictory." In a Bayesian hierarchical model, variation across groups is a finding to be modeled and explained, not a contradiction to be resolved.

- Treat the variation in β parameters across contexts as a result in itself.
- Use the posterior distributions to compare these contexts directly. For instance, what is the probability that the effect in the Private Company is *larger* than in the University? The Bayesian framework allows for direct probabilistic comparisons between groups ($P(\beta_{company} > \beta_{university})$).

Using this metric would substantiate the claims about organizational structure more effectively than looking at separate CIs.

3. **Visual Presentation of Uncertainty**

Figures 1 and 2 display the median and CIs. Because the authors discuss the "probability" of effects in the text (e.g., "probability of 80% that PR is below one" 3), the figures should aid this interpretation.

- Ensure the figures clearly communicate the density or the spread of the posterior, if possible, or at least ensure the captions explain the meaning of the intervals in probabilistic terms rather than frequentist significance.
- Update the y-axis labels. Codes like W-W/Mix are hard to parse. Use full descriptors such as "Women Homophily" or "Same-Gender Preference."
