# Supplementary material for: Gender, status, and team interaction: A microdynamic exploration of wearable sensor data across 11 research groups
Source: PLoS One. 2026 May 15;21(5):e0349195. doi: 10.1371/journal.pone.0349195 (PMC13178976; doi:10.1371/journal.pone.0349195)
Supplement: Attachment — Submitted filename: Review_PONE-D-25-13840_R2.pdf [file pone.0349195.s007.pdf]

Manuscript Number: PONE-D-25-13840R2

Gender, Status, and Team Interaction: A Microdynamic Exploration of Wearable Sensor Data Across 11 Research Groups

The authors have engaged carefully with the previous round of comments, and the revisions address the core concerns raised. The paper can now be recommended for acceptance, with a few observations noted below for the authors' consideration.

1. Response to previous comments

The three main points raised in the previous review concerned: (i) the underutilization of the Bayesian framework through reliance on binary significance language; (ii) the characterization of cross-context variation as "contradictory" rather than as a substantive finding; and (iii) the visual presentation of uncertainty in the figures.

All three have been addressed satisfactorily. The authors now report the probability of direction (pd) alongside credible intervals and use it to construct a graduated evidence scale (strong, moderate, weak, insufficient). This is a meaningful improvement. The treatment of cross-context variation has also improved. The figures have been updated to half-eye plots displaying the full posterior density alongside the 50% and 95% CIs, and the axis labels now use readable descriptors.

2. Coherence of the empirical story

The paper's empirical story is coherent, and the results are broadly consistent with the hypotheses as reformulated under the Bayesian framework. Three observations follow.

H1 (gender homophily). The finding that same-gender dyads, particularly women-women, are less likely to interact than mixed-gender dyads in universities and research labs is clearly supported and consistently presented. The contrast with the private company context, where evidence is insufficient, is now handled as a substantive heterogeneity finding rather than an anomaly to be explained away. This is the right framing.

H2 (professional status). The results for H2 are weak and context-dependent, and the authors acknowledge this directly. The finding that senior-senior dyads are approximately one-third as likely to interact in private companies, in contrast to the weakly positive evidence in universities, is consistent with the argument that flat organizational structures reduce the salience of formal status hierarchies as cues for interaction. The discussion section draws this contrast effectively.

H3 (gender-based status cues). The most striking finding is the near-reversal between research labs and private companies for H3b. In research labs, women-women senior dyads are approximately half as likely to interact as mixed-gender senior dyads (strong evidence), whereas in private companies, the same dyad is estimated to be approximately 25 times more likely to interact (strong evidence, very wide CI). The authors interpret this through the "chilly climate" hypothesis and the gender-status mismatch literature. This interpretation is plausible, though the private-company estimate is extremely uncertain (95% CI [1.58, 200]). The discussion appropriately qualifies the private-company result on these grounds. The contrast itself is real and well-supported by the $\Delta\beta$ comparison (pd = 1.00).

3. Remaining minor points

These observations may require minor further revision.

The inertia result deserves a brief comment in the discussion.

Negative inertia is estimated across all three contexts, meaning that past interactions do not increase the probability of future ones. In a setting where recency is very strongly positive, this pattern likely reflects the structure of the event history: consecutive detections within a session are aggregated, so inertia and recency may be capturing distinct time scales (session-to-session vs. within-session). The authors mention that the strong recency effect is not a sensor artifact, but they do not note the potentially puzzling conjunction with negative inertia. A sentence acknowledging this dynamic would strengthen the discussion.

The probability ratio for private companies under H3b is extremely wide.

A PR of 25 with a CI of [1.58, 200] is a valid posterior summary, but readers unfamiliar with Bayesian reporting may interpret the point estimate as more informative than it is. The figure caption notes that the posterior has been truncated for the private company; the supplementary file provides the numerical values. It would be worth adding a brief in-text

note flagging the high uncertainty of this estimate specifically, beyond the general acknowledgment in the limitations section.

The "chilly climate" interpretation is one of two plausible readings.

An alternative explanation for the private-company pattern, consistent with the data and with the literature, is that mixed-gender senior interaction in flat organizations is lower because gender-based status differences are more salient in the absence of formal hierarchy, prompting avoidance of cross-gender contact for senior women rather than women's mutual support as the primary mechanism. The authors gesture at this in the discussion but do not distinguish clearly between the two mechanisms. Given that both are consistent with the observed pattern, the discussion could note the ambiguity explicitly rather than privileging the chilly-climate reading.

4. Recommendation

The manuscript has improved substantially across three rounds of review. The methodological concerns raised previously have been addressed, the Bayesian framework is now used consistently, and the empirical story is presented with appropriate acknowledgment of uncertainty and context-dependence. The paper makes a genuine contribution to the team diversity literature by providing one of the first hierarchical DyNAM analyses of real-world face-to-face interaction data, and by demonstrating that gender and status effects are strongly context-dependent.
