# Supplementary material for: Gender, status, and team interaction: A microdynamic exploration of wearable sensor data across 11 research groups
Source: PLoS One. 2026 May 15;21(5):e0349195. doi: 10.1371/journal.pone.0349195 (PMC13178976; doi:10.1371/journal.pone.0349195)
Supplement: Attachment — Submitted filename: Reply-to-Reviewers-3rd-submission.docx [file pone.0349195.s006.docx]

**We would like to thank the anonymous reviewer for the suggested improvements. In what follows, we describe how each point has been addressed.**

**Editors feedback:**

[1] Report the Probability of Direction (pd). Calculate and report the percentage of the posterior distribution that shares the same sign as the median. This provides a direct probability statement (e.g., "there is a 93% probability that gender homophily is positive") which is more intuitive and accurate than a CI-based rejection.

*REPLY: results reporting includes now both CI and pd values.*

*“To interpret the fitted models, we summarise the posterior distributions using the median as a point estimate and the 95% equal-tailed credible interval (CI), which contains the central 95% of the posterior mass and excludes the lowest and highest 2.5%. We also report the probability of direction (pd), which quantifies directional certainty by computing the proportion of posterior samples that share the same sign as the median [149].*

*Hypotheses are evaluated by jointly considering the probability of direction and the credible intervals. We classify evidence strength as follows: strong directional evidence when the 95% CI excludes the reference value (zero for β coefficients, one for probability ratios), this necessarily implies pd ≥ 0.975; moderate directional evidence when pd ≥ 0.95; weak directional evidence when pd ≥ 0.90; and insufficient directional evidence when pd < 0.90. Together, these metrics indicate whether an effect is likely to exist and provide its plausible range, allowing us to assess the strength of evidence for each hypothesis.”*

[2] Discuss Magnitude and Uncertainty. Instead of simply accepting or rejecting hypotheses, discuss the strength of the evidence provided by the posterior. If 90% of the posterior mass is positive, this is worth discussing as a probable effect, even if the 95% CI includes zero.

*REPLY: We discuss now both magnitude and uncertainty in the methods section and propose to qualify the obtained evidence as strong, moderate or no evidence based upon a combination of CI and pd values.*

*For example: “]). The effect is stronger in research labs, where women-women dyads are approximately one-third as likely to interact next (β₅ˡᵃᵇ = –0.54, 95% CI [–0.75, –0.33], pd = 1.00; PRˡᵃᵇ = 0.34, 95% CI [0.22, 0.51]). Private companies show insufficient evidence of any effect (β₅ᵇᵘˢ = 0.17, 95% CI [–1.18, 1.21], pd = 0.61; PRᵇᵘˢ = 1.40, 95% CI [0.09, 11.20]).”*

[3] Reformulate Conclusions. Re-evaluate the "rejected" hypotheses (e.g., H2a, H3a) using these probabilistic metrics. It is likely that the data provides stronger support for your theoretical claims than the current binary interpretation suggests.

*REPLY: We have removed statements regarding a clear “acceptance” or “rejection” of stated hypothesis but discuss our findings more in alignment with Bayesian approach.*

[4] Treat the variation in beta-parameters across contexts as a result in itself and use the posterior distributions to compare these contexts directly.

*REPLY: We have furthermore incorporated the variation across contexts in our model and results, indicating where beta-parameters differ, the strength of the evidence (CI and pd-base).*

*“To assess organisational heterogeneity, we report posterior statistics for the largest pairwise difference in β coefficients between contexts (Δβ), along with 95% CIs and probability of direction. These direct comparisons quantify whether observed cross-context differences are substantive and provide their plausible range.”*

*For example, when reporting results: “The organisational contrast between private companies and universities shows insufficient evidence of a substantial difference (Δβ₅^bus-uni^ = 0.52, 95% CI [-0.86, 1.61], pd = 0.79).”*

[5] Visual presentation: Ensure the figures clearly communicate the density or the spread of the posterior.

*REPLY: We have revised the provide illustrations.*

*“Figure 1 displays half-eye plots of the posterior distribution for the PRs of gender homophily by seniority level. The upper half shows the posterior densities; the lower half displays summary statistics: the median (dot), the 50% equal-tailed credible interval (thick line), and the 95% equal-tailed credible interval (thin line). This visualization allows inspection of both the full posterior and conventional interval-based summaries.”*

[6] Update the y-axis labels.

*REPLY: Axis labels have been updated and include now full descriptions.*
